# Robot-assisted needle insertion for venous catheterization

**DOI:** 10.1590/S1679-45082015MD3374

**Published:** 2015

**Authors:** Jacyr Pasternak

**Affiliations:** 1 Hospital Israelita Albert Einstein, São Paulo, SP, Brazil.

**Keywords:** Robotics, Artificial intelligence, Vascular access devices/trends, Catheterization, central venous/instrumentation, Catheterization, central venous/methods

## Abstract

Vein access can be challenging for a variety of patients. The development of robots-assisted central or peripheral veins puncture would facilitate life of health professionals and patients. New robots are under development for this purpose and probably they will become available for practical use in the near future. These techniques may decrease significantly the cost of medicine, which currently uses less informatics resources than other industries.

Ultrasound has been employed routinely for intravenous catheter insertion at our institution and it has increased the success rate of insertion. Venous location also has been made easier with other systems such as special illumination. In addition, more complete robotic devices are under development for needle invasive procedures, such as breast biopsy,^(^ ^1^ ^)^ percutaneous renal access^(^ ^2^ ^)^ or cholecystostomy;^(^ ^3^ ^) ^and central venous catheterization. Currently, the problems involved with the use of these devices have been addressed and published in the literature.^(^ ^4^ ^) ^Everyday, many intravenous catheters and venous punctures are done, and they are highly operator dependent. Venous access can be difficult, painful and lead to severe stress in cancer patients who had undergone chemotherapy, obese patients and children.

The trypanophobia, dread of needles, can be idiopathic (the individual never performed a venipuncture, but faints at the sight of a needle) or acquired. There are many reasons for the acquired form of this phobia, such as high failure rates to vein puncture (25 to 30%), need of another puncture, and the increased failure rate because of screaming children and non-cooperative individuals.

A recent study is bringing these devices for venous access to clinical practice. In addition, a collaborative research between Ben-Gurion University of the Negev and Cincinnati Children Hospital designed a prototype device that integrates ultrasound and a TV camera with a robotic needle-dispenser. A human operator is still needed, but what he does is easy: tracks the vein, aligns a target icon over it, and the robotic system does the rest. The operator has only to push a button when absolutely sure that the icon is over a vein.^(^ ^5^ ^)^

Another device, already named, is the Hemobot designed by Salisbury Robotics, which is almost ready to undergo clinical trials,^(^ ^6^ ^)^ Besides this, other pre-commercial devices are under development and they will be launched soon.

The probable impact of these devices, if they come to clinical practice, should be remarkable. Vein access is time-consuming for technicians working in clinical laboratories and nurses. This device may minimize the time spent by these professionals in these chores and this extra time could be used to improve nursing care. It would be an important step if results in less expensive medical care. One of the main critics of health professionals is that new resources and informatics do not impact as expected in health care cost reduction. Perhaps when devices like these reach the patient’s bedside, cost of medicine will go down.
