# Supplementary material for: Robot-assisted needle insertion for venous catheterization
Source: Einstein (Sao Paulo). 2015 Jul-Sep;13(3):475–6. doi: 10.1590/S1679-45082015MD3374 (PMC4943800; doi:10.1590/S1679-45082015MD3374)
Supplement: Translation

# Translation: Injeção-robô para cateterismo venoso

**Authors:** Jacyr Pasternak

**Affiliations:** 1 Hospital Israelita Albert Einstein, São Paulo, SP, Brasil.

**Keywords:** Robótica, Inteligência artificial, Dispositivos de acesso vascular/tendências, Cateterismo venoso central/instrumentação, Cateterismo venoso central/métodos

## Abstract

O acesso venoso pode ser difícil em muitos pacientes. O desenvolvimento de robôs com capacidade de puncionar veias centrais ou periféricas pode facilitar a vida dos profissionais de saúde e dos pacientes. Novos robôs estão sendo desenvolvidos para essa finalidade e, provavelmente, entrarão em uso na prática clínica em um futuro próximo. Técnicas como estas poderão baixar progressivamente o custo em medicina, que, atualmente, tem usado menos recursos de informática que outras áreas.

Rotineiramente, nossa instituição tem utilizado o ultrassom para inserção de cateter intravenoso, fato que tem aumentado à taxa de sucesso do procedimento. A localização venosa também tem sido facilitada com outros sistemas como, por exemplo, a iluminação especial. Além disso, dispositivos-robôs mais completos estão em desenvolvimento para uso em procedimentos invasivos, como a biópsia da mama,^(^ ^1^ ^)^ o acesso renal percutâneo^(^ ^2^ ^)^ ou colecistostomia;^(^ ^3^ ^)^ e a cateterização venosa central. Atualmente, os problemas envolvidos no uso desses dispositivos têm sido abordados e publicados na literatura.^(^ ^4^ ^) ^Todos os dias, diversos cateteres intravenosos e punções venosas são realizadas, e eles são procedimentos altamente dependentes de operador. O acesso venoso pode ser difícil e doloroso aos pacientes oncológicos submetidos a quimioterapia, obesos e pediátricos. Além disso, pode causar estresse crônico nesses indivíduos.

A tripanofobia, medo irracional a procedimentos que envolvam agulhas, pode ser idiopática (indivíduo que nunca realizou uma punção venosa, mas que pode desmaiar ao ver uma agulha) ou adquirida. Diversas razões são apontadas para a forma adquirida dessa fobia, como altas taxas de falha da punção venosa (25 a 30%), e necessidade de nova punção, aumento da taxa de falha devido à agitação da criança e indivíduos que não cooperam durante a punção.

Estudo recente sobre acesso venoso leva esses procedimentos automatizados para a prática clínica. Além disso, uma pesquisa colaborativa entre a *Ben-Gurion University of the Negev* e *Cincinnati Children Hospital* desenvolveu um protótipo que integra ultrassom, câmera plugada a uma TV e dispensador robô de agulha. Contudo, um operador humano ainda é necessário, mas o que ele precisa fazer é muito simples: localizar a veia, e alinhar o ícone sob a veia; o sistema robótico faz o resto. Para punção, o operador precisa apenas apertar um botão quando seguro de que o ícone está sob a veia.^(^ ^5^ ^)^

Um outro dispositivo, já com nome comercial, o *Hemobot*, foi desenvolvido pela *Salisbury Robotic* e já está quase pronto para testes em ensaios clínicos.^(^ ^6^ ^)^ Além deste, outros equipamentos parecidos estão em fase pré-comercial e serão lançados em breve.

O impacto desses dispositivos, se chegarem na prática clínica, será de grande importância. O acesso venoso consome uma grande quantidade de horas do trabalho dos técnicos que atuam em laboratórios clínicos e de enfermeiros. Esses dispositivos podem reduzir o tempo dispendido por esses profissionais nessa atividade, sendo que o tempo extra conquistado pode ser aproveitado para melhoria da assistência de enfermagem. Trata-se de passo importante, caso resulte em redução de custo. Uma das principais críticas dos profissionais de saúde é que novos recursos e a informática não impactam como esperado na redução dos custos na assistência médico-hospitalar. Talvez quando esses dispositivos atingirem a beira do leito, uma real redução de custo em medicina poderá ser alcançada.
